# Association between serum 25-hydroxyvitamin D concentrations and hypertension among adults in North Sudan: a community-based cross-sectional study

**DOI:** 10.1186/s12872-023-03432-3

**Published:** 2023-08-17

**Authors:** Ahmed A. Hassan, Omer Abdelbagi, Osman E. Osman, Ishag Adam

**Affiliations:** 1 Faculty of Medicine, University of Khartoum, Khartoum, Sudan; 2 Department of Pathology, AlQunfudhah Faculty of Medicine, Umm Al-Qura University, Al Qunfudhah, Saudi Arabia; 3 Faculty of Medicine, Alneelain University, Khartoum, Sudan; 4 Department of Obstetrics and Gynecology, Unaizah College of Medicine and Medical Sciences, Qassim University, Unaizah, Saudi Arabia

**Keywords:** Hypertension, Systolic, Diastolic, Vitamin D, Sudan

## Abstract

**Background:**

Globally, hypertension represents a major public health problem. The association between 25-hydroxyvitamin D (25[OH]D) levels and hypertension remains unclear. The current study aimed to investigate the association between serum 25(OH)D levels and hypertension among adults in Sudan.

**Methods:**

A community-based cross-sectional study was conducted among adults in North Sudan. Sociodemographic and clinical data were collected using a questionnaire and face-to-face interviews. Serum 25(OH)D was measured using an enzyme-linked immunosorbent assay. Multivariate logistic regression and multiple linear regression analyses were performed.

**Results:**

Of the total of 391 participants, 202 (51.7%) were females. The median (interquartile range [IQR]) of participants’ ages was 45(32–55) years. Of the total, 219(56.0%) had hypertension. The median (IQR) of serum25(OH)D was 13.3(9.9–19.7) ng/mL, and 295 (75.4%) participants had vitamin D deficiency (< 20 ng/mL). In multivariable logistic regression, the adjusted odds ratio (AOR) for age = 1.05, 95% confidence interval (CI)1.03‒1.061, the AOR for being female = 2.02, 95% CI, 1.12‒3.66, and body mass index was AOR = 1.09, 95% CI, 1.05‒1.14, all of which were significantly associated with hypertension. However, serum 25(OH)D levels were not associated with hypertension (AOR = 1.01, 95% CI 0.99‒1.05, *P* = 0.317). In multiple linear regression, while systolic blood pressure was negatively associated with 25(OH)D (coefficient = − 0.28, *P* = 0.017), there was no significant association between serum 25(OH)D level and diastolic blood pressure (coefficient = − 0.10, *P* = 0.272) or mean blood pressure (coefficient =–0.03, *P* = 0.686).

**Conclusion:**

The current study revealed a negative association between vitamin D and systolic blood pressure. The mechanism of such an association needs further study.

## Introduction

Hypertension is a global health problem among adults (31.1% of those aged ≥ 20 years) and is a leading cause of global morbidity and mortality [1].The burden of hypertension is much higher in resource-limited settings such as Sub-Saharan Africa, where approximately 30% of the population has hypertension [2]. Several factors, such as age, gender, and obesity, have been reported as risk factors for hypertension [3]. Vitamin D is a fat-soluble vitamin that plays an important role in health [4]. Several diseases, such as hypertension, obesity and obesity-related diseases, cardiovascular and cerebrovascular diseases, type 2 diabetes, Alzheimer’s disease, and schizophrenia, are associated with vitamin D [5–9]. Vitamin D deficiency is a major worldwide health problem, especially in African countries [10].

While some studies have shown a significant association between vitamin D and hypertension [6, 8, 11–14], others have found no association [15, 16]. Such uncertainty in data regarding the association between vitamin D deficiency and hypertension necessitates further research, especially in understudied settings with limited resources. Moreover, the heavy burden of vitamin D deficiency on health requires urgent public health action [17]. Therefore, the practical steps for addressing vitamin D deficiency and its complications on a global scale require a thorough understanding of the local context. To achieve this, first, the prevalence of vitamin D deficiency in the community, in addition to its association with blood pressure level, needs to be investigated, and then, appropriate healthcare measurements can be applied accordingly. Sudan shows a high prevalence of hypertension among adults [18]. However, the association between vitamin D and hypertension has not been investigated in Sudan. Hence, such an association needs to be explored, aiming to address both public health problems (vitamin D deficiency and hypertension). The current study aimed to investigate the association between serum 25(OH)D levels and hypertension in northern Sudan.

## Materials and methods

### Study area

River Nile is one of the 18 states of Sudan. Based on the 2008 census, its total population was 1,120,441 [19]. There are seven localities (the lowest administrative unit in Sudan) in the River Nile state.

### Study population and design

This community-based cross-sectional study was conducted in October and November 2021in four villages in the Wad Hamid district, Almatamah locality, River Nile State, northern Sudan, which is adjacent to Khartoum State and about 150 km from Khartoum, the capital of Sudan. Strengthening the reporting of observational studies in epidemiology (STROBE) guidelines were strictly followed [20].

Initially, one locality, Almatamah, was randomly selected from seven. From the three districts of the Almatamah locality, Wad Hamid was selected randomly. Four villages (Hajer Alteer, Athawra Kabota, Wadi Alshohda, and Alkoumer) were chosen from the randomly selected district using a systematic sampling method. Then,30 to 40 households from each village were selected based on population density to reach the desired sample size (*n* = 391). The first member in each household who agreed to participate and met the study inclusion criteria was selected. If the selected house was uninhabited or the inhabitants declined to participate, the next house was selected to complete the target number for the study. The investigators trained four medical officers in data collection methods. After signing an informed consent form, all adult (aged ≥ 18 years) Sudanese residents (both men and women) were enrolled from households chosen using a lottery method. Participants under age 18 years, pregnant women, people with poor cognitive functions, and severely ill people were excluded from this study.

### Data collection

The World Health Organization’s (WHO) three-level stepwise approach questionnaire [21] was used for data collection. The questionnaire gathers sociodemographic data, including age in years, gender, marital status (married/unmarried), education level (< secondary and ≥ secondary), and employment status (employed/unemployed).

### Procedures

Blood pressure was measured using an appropriate cuff size with a standard mercury sphygmomanometer after resting for at least 10 min in a sitting position, with the arm maintained at heart level. The mean of two blood pressure readings taken at 1–2 min intervals was recorded. If the difference between the two was > 5 mmHg, measurements were retaken until the reading stabilized.

The participant’s weight was measured in kg using standard procedures (well-calibrated scales adjusted to zero before each measurement). The participants stood with minimal movement, with hands by their side. Moreover, shoes and excess clothing were removed. Then, height was measured in cm after being made to stand straight with the participant’s back against the wall and the feet together. The body mass index (BMI) was computed as the weight in kg divided by the square of the height in meters (kg/m^2^). The BMI was categorized according to the WHO classification as underweight (< 18.5 kg/m^2^), normal weight (18.5–24.9 kg/m^2^), overweight (25.0–29.9 (kg/m^2^), and obese (≥ 30.0 kg/m^2^) [22]. Participants were considered hypertensive based on prior diagnosis and the use of antihypertensive medications or their average systolic blood pressure was ≥ 140 mmHg, or average diastolic blood pressure was ≥ 90 mmHg, or with both criteria met [23].

### Processing of blood samples

Five milliliters of blood was collected from the cubital vein into a plain tube and allowed to clot at room temperature. The blood was then centrifuged and stored at − 20° C until we performed the assay of 25(OH)D using the enzyme-linked immunosorbent assay (fully automaTable 450 nm, reference wavelength between 620 and 650 nm) following the manufacturer’s instructions (Euroimmun, Lubeck, Germany). Manufacturer quality control measures and 6 levels of standard solutions (calibrator) set between 0 and 120 ng/mL were applied for each assay. The sample was considered vitamin D deficient if the serum 25(OH)D level was < 20 ng/mL [24].

### Sample size calculation

A total of 384 participants were included in this study as (*n*) with an assumed prevalence of hypertension of 50%,theprevalence previously reported in eastern Sudan [3], using a single proportional formula (*n* = Z^2^pq/ d^2^), Q = (1-p), Z1-α = CI 95% = 1.96, d = margin of error of 5% = 0.05.Werounded up and concluded that the sample size should be 391 participants to allow for missing or inadequate samples.

### Statistical analysis

Data were entered into the IBM Statistical Package for the Social Sciences® (SPSS®) for Windows, version 22.0 (SPSS Inc., New York, United States). The proportions were expressed as frequencies (%). Continuous data were evaluated for normality using the Shapiro–Wilk test and were non-normally distributed. The non-normally distributed data were expressed as a median (interquartile range [IQR]). Adjusted regression analysis (multivariate and multiple linear) was performed on hypertension (for multivariate), systolic, diastolic, and mean blood pressure (multiple linear regression) as dependent variables, sociodemographics (age, gender, BMI, educational level, marital status, and occupation) and25(OH)D levels as independent variables. Adjusted odds ratios (AORs), 95% confidence intervals (CIs), coefficients, and standard errors were calculated as they were applied. A two-sided *P*-value of < 0.05 was considered statistically significant.

## Results

A total of 391participants were enrolled in this study. Two hundred two (51.7%) participants were women, and the remaining 189 (48.3%) were men. The median (IQR) of participants’ age was 45 (32–55) years. Of the 391 enrolled participants, 219 (56.0%) had hypertension, 68 of whom (17.4%) had been previously diagnosed and 151 (38.6%) were newly diagnosed.

The median (IQR) of participants’ serum 25(OH)D was 13.3(9.9–19.7) ng/mL. Of the total 391, 295 (75.4%) had vitamin D deficiency. The levels of deficiency among hypertensive and non-hypertensive participants were 163 (74.4%) and 132 (76.7%), *P* = 0.598, respectively.

The multivariable logistic regressions for age (AOR = 1.05, 95% CI1.03‒1.06), being female (AOR = 2.02, 95% CI 1.12‒3.66), and BMI (AOR = 1.09, 95% CI 1.05‒1.14) were significantly associated with hypertension. However, the serum 25(OH)D level was not associated with hypertension (AOR = 1.01, 95% CI 0.99‒1.05, *P* = 0.317) (Table 1). In multiple linear regression, there was no association between serum 25(OH)D level and diastolic blood pressure (coefficient = 0.10, *P* = 0.272) or mean blood pressure (coefficient = − 0.03, *P* = 0.686). Only systolic blood pressure was negatively associated with serum 25(OH)D level (coefficient = − 0.28, *P* = 0.017) (Table 2).

**Table 1:** Univariate and multivariate analyses of the factors associated with hypertension

Variable		Participants with hypertension (*n* = 219)	Participants without hypertensive (*n* = 172)	Univariate analysis		Multivariate analysis	
		Range (interquartile range)		OR (95% CI)	*P*	OR (95% CI)	*P*
Age, years		50.0(38.0‒60.0)	38.0(28.0‒50.0)	1.04(1.02‒1.05)	< 0.001	1.05(1.03–1.106)	< 0.001
Body mass index, kg/m^2^		27.5(24.0‒31.3)	24.3(19.4‒28.3)	1.11(1.07‒1.15)	< 0.001	1.09(1.05–1.14)	< 0.001
25-hydroxyvitaminconcentration (ng/mL)		13.6(9.8–20.4)	12.6(10.0–19.3)	1.01(0.98–1.03)	0.701	1.012(0.99–1.05)	0.317
		Frequency (proportion)					
Gender	Male	92(42.0)	97(56.4)	Reference			0.020
	Female	127(58.0)	75(43.6)	1.79(1.19‒2.67)	0.005	2.02(1.12–3.66)	
Education level	≥ secondary	149(68.0)	118(68.6)	Reference			0.552
	<secondary	70(32.0)	54(31.4)	1.03(0.67‒1.58)	0.905	1.16(0.72–1.88)	
Occupational status	Employed	89(40.6)	83(48.3)	Reference			0.857
	Unemployed	130(59.4)	89(51.7)	1.36(0.91‒2.04)	0.132	0.95(0.53–1.71)	
Marital status	Married	45(20.5)	54(31.4)	Reference			0.673
	Unmarried	174(79.5)	118(68.6)	1.77(1.12‒2.80)	0.015	0.89(0.51–1.55)	

**Table 2:** Linear regression analysis of factors associated with systolic, diastolic, and mean blood pressure

Variable		Systolic blood pressure		Diastolic blood pressure		Mean blood pressure	
		Coefficient (standard error)	*P*	Coefficient (standard error)	*P*	Coefficient (standard error)	*P*
Age, years		0.34(0.06)	< 0.001	0.14(0.04)	0.003	0.20(0.04)	< 0.001
Body mass index, kg/m^2^		0.18(0.14)	0.236	0.42(0.11)	< 0.001	0.34(0.09)	0.001
25-hydroxyvitamin D concentration (ng/mL)		–0.28(0.12)	0.017	0.10(0.09)	0.272	–0.03(0.08)	0.686
Gender	Male	Reference					
	Female	1.82(2.40)	0.449	1.10(1.77)	0.534	1.34(1.56)	0.390
Education level	≥ secondary	Reference					
	<secondary	–4.17(1.97)	0.035	1.76(1.45)	0.224	–0.22(1.28)	0.866
Occupational status	Employed	Reference					
	Unemployed	–0.10(2.39)	0.967	–63(1.75)	0.722	–0.45(1.55)	0.771
Marital status	Married	Reference					
	Unmarried	0.59(2.26)	0.793	2.72(1.66)	0.102	2.01(1.46)	0.170

## Discussion

In the current study, serum 25(OH)D was inversely associated with systolic blood pressure. The inverse association between vitamin D deficiency and systolic blood pressure is in agreement with a previous study conducted in Iran [25] in which a prospective population-based cohort study among 9,520 adults reported a significant negative association between serum 25(OH)D levels and systolic blood pressure (β = − 0.02, *P* = 0.04) [25].A recent study conducted in southern India investigated the association between 25(OH)D levels and hypertension in the general population (400 hypertensive vs. 400 normotensive) and showed that serum vitamin D deficiency was significantly associated with both mean systolic and mean diastolic blood pressure [11].

This study found no significant association between serum 25(OH)D levels and hypertension. Similarly, a large survey in Saudi Arabia that included 957 women and 1,127 men showed no evidence of an association between serum25(OH)D levels and hypertension [26]. In contrast, other studies revealed that serum 25(OH)D concentrations were inversely related to the risk of hypertension in adults [12, 27]. Moreover, a recent meta-epidemiological analysis conducted by Bae attributed a significant association between circulating 25(OH)D levels and the risk of hypertension [28].

Other studies have found that serum25(OH)D was inversely associated with hypertension [6, 8, 12–14]. In Turkey, a recent study conducted by Karadeniz et al. followed 491 healthy middle-aged participants without any chronic illness and showed low levels of 25(OH)D associated with the development of hypertension in an 8-year follow-up [12]. Interestingly, in the current study, only systolic blood pressure (no association with hypertension, diastolic, or mean blood pressure) was associated with vitamin D deficiency. This unique association between vitamin serum 25(OH)D and systolic blood pressure could be explained by the influence of vitamin D on cardiac contractility via calcium. Hence, vitamin D deficiency leads to a decline in calcium absorption by the intestines (hypocalcemia) [29]. Further research is recommended to better understand the mechanisms of such a unique association. Furthermore, the very low serum 25(OH)D in the current study (15.5 ± 7.8 ng/mL) compared to previous studies in Iran (21.75 ± 12.3 ng/mL) [25] and Pakistan (35.99 ± 8.08 ng/mL) [30] suggests that further vitamin D deficiency epidemiological studies are needed. Recently, there has been ongoing debate among experts regarding the definition (cut-off point)of vitamin D deficiency [31]. A recent study that merits mention was conducted in Qatar to examine the issue of justifying the use of the existing cut-off value and called for defining a new cut-off value for Qatar [8].

Our results should be compared cautiously with findings to the contrary in previous studies. First, our study was conducted in asymptomatic participants in a community, while some other results were from symptomatic participants or facility-based studies. Second, differences in the heterogeneity of participants’ baseline sociodemographic characteristics, such as age, should be taken into account. Third, there were differences in sample size among the studies. Fourth, different hypertension parameters have been used across the studies (i.e., hypertension, mean systolic, mean diastolic, mean blood pressure, and isolated systolic/diastolic).

The exact mechanism by which vitamin D may influence blood pressure is not well known; however, several possible explanations have been posited by researchers to explain the association between vitamin D and hypertension, such as the key influential role of vitamin D on the metabolism of lipid and trace minerals (calcium, phosphate, and magnesium) [11, 30, 32]. Such lipid profiles and trace minerals have an impact on blood pressure status [32, 33].The anti-inflammatory effects of vitamin D may mitigate the development of hypertension [34]. Zhou and Hypponen, observed an association between 25(OH)D and C-reactive protein (CRP) levels, and they attributed the cause to vitamin D deficiency [35]. Moreover, they recommended correcting low vitamin D status to reduce chronic inflammation [35]. In their family-based study, Bai et al. attributed the association between vitamin D deficiency and hypertension to genetic factors [13].The current study was conducted primarily to assess the association between vitamin D and hypertension. As shown in the regression analysis, many other factors, such as age, gender, and BMI, are associated with hypertension. We have previously discussed these issues in our previous work in eastern Sudan [3]. Furthermore, a discussion of these factors might extend this manuscript.

### Limitations

The nature of the present study, which was cross-sectional, did not differentiate causes or effects. A longitudinal study will provide more clarity regarding an association between vitamin D and hypertension. This study covered one geographical area; thus, it may not represent the entire country (Sudan). Some factors, such as physical inactivity, dietary factors, and blood glucose levels, were not explored in this study. The lipid profile and CRP were not assessed in the current study.

## Conclusion

The current study found that three-quarters of the participants were vitamin D deficient. There was a significant negative correlation between vitamin D and systolic blood pressure. The mechanism of such an association warrants further study.

## Data Availability

The datasets generated and/or analyzed during the current study are not publicly available (the manuscript is still under peer review) but are available from the corresponding author upon reasonable request.

## Abbreviations

25(OH)D: 25-hydroxyvitamin D
BMI: Body mass index
CI: Confidence interval
cm: Centimeter
IQR: Interquartile range
kg: Kilogram
m: Meter
SPSS: Statistical Package for the Social Sciences
STROBE: Strengthening the reporting of observational studies in epidemiology
WHO: World Health Organization
